# Current limitations of exercise snacks studies: A road map for the future

**DOI:** 10.17179/excli2026-9491

**Published:** 2026-06-19

**Authors:** Fatemeh Sadat Masoudi, Farhad Daryanoosh, Kayvan Khoramipour

**Affiliations:** 1 Department of Sport Science, Faculty of Education and Psychology, Shiraz University, Shiraz 71946-84759, Iran; 2 i+HeALTH Strategic Research Group, Department of Health Sciences, Miguel de Cervantes European University (UEMC), 47012, Valladolid, Spain

## ⁯⁯

The beneficial effects of physical activity (PA) on health and well-being have been recognized for centuries. Greek philosopher Plato, for instance, referred to the necessity of PA by stating that “lack of activity destroys the good condition of every human being, while movement and PA improve or at least preserve it” (Tipton, 2014[21]). Research showed that PA could counteract cardiovascular disease (Luo et al., 2024[13]), type 2 diabetes (Gallardo-Gómez et al., 2024[7]), cancer (Yang et al., 2024[26]), neurodegenerative diseases (Ben Ezzdine et al., 2025[3]), obesity (Xu et al., 2024[25]), and many other chronic diseases. In addition to physical benefits, PA brings a lot of mental advantages, such as reducing anxiety, stress, depression, and improving self-esteem, positivity, and life expectancy (White et al., 2024[24]). Nevertheless, modern lifestyles have shifted from being physically active to being more machine-dependent and sedentary. Continuing this trend will lead to the global physical inactivity cost of around 500 billion dollars between 2020 and 2030 (Pettican et al., 2024[16]).

The most cited reasons for avoiding PA are work commitments, lack of motivation, time constraints, high costs, limited access to sports facilities, and fear of injury (Luo et al. 2024[13]). Seeking to overcome these barriers, researchers developed the concept of exercise snacks (ESs). The idea is simple: instead of saving all your energy for one workout at the end of the day (a session that, realistically, is often postponed or skipped), you incorporate a microdose of PA into your daily routine. Each ES session could last between 20-720 seconds and could be done as many times as possible during the day (Weston et al., 2025[23]). However, the interplay of session duration, intensity, and frequency should be considered to ensure adherence to the American College of Sports Medicine recommendation (i.e., 150 minutes of moderate-intensity or 75 minutes of high-intensity exercise training per week) (Alabdul Razzak et al., 2025[1]).

This method may include structured exercises such as endurance training (e.g., stair climbing), resistance training (e.g., body-weight exercises), or a combination of both (Brandt et al., 2024[4]; Yin et al., 2024[27]). Regular activities such as brisk walking over short intervals also fall under this model. The most distinguishing feature of this model is that it is time-efficient and requires no specialized equipment, making it a convenient and practical solution to combat the causes associated with a lack of PA. Thus, ESs can be a practical substitute for those unable or unwilling to participate in traditional, prolonged exercise sessions (Jones et al., 2024[9]; Wang et al., 2025[22]).

Schmidt et al. (2001[19]) used the ESs protocol in 2001, but in 2006, Elley et al. (2006[5]) formally introduced the term ESs. The second study was published in 2014 by Francois et al. (2014[6]) and the third in 2016 (Perkin et al., 2016[15]). 2018 and 2019 were a turning point for ESs studies with 5 publications. Between 2020 and 2025, 43 studies have been published, bringing the total number of publications to 51, showing a strong upward trend in recent years (Supplementary Figure 1). While these studies provided useful data regarding ESs, they have some limitations.

The first and the most important one is that they exclusively focus on clinical outcomes such as body weight, blood glucose, blood pressure, and lipid profiles, neglecting the deep mechanistic approach. Although such findings are informative, they limit our understanding of the exact mechanisms of ESs effects. To overcome this limitation, high-throughput untargeted omics such as Enduromics and Resistomics can be employed to quantify thousands of molecules and provide global insights into their functional roles (Khoramipour et al., 2025[10]). Furthermore, omics studies could be done in biofluids and do not need invasive tissue sampling. In addition to omics-based approaches, an important avenue is using animal models. Animal models offer a valuable opportunity to conduct tissue-specific analyses in a systematic, multilayered, and controlled environment. In addition, animal models allow researchers to evaluate biological outcomes across multiple tissues (e.g., skeletal muscle, adipose tissue, liver, and heart) and inter-tissue associations under strictly controlled experimental conditions (Khoramipour et al., 2022[11]). Without such data about the molecular transducers of ES, we would not be able to individualize ES programs, as knowing the mechanism of action is the most important and primary step in designing an exercise plan specifically for anyone.

Robust methodological design is another important step in studying the molecular mechanisms of ES adaptations. Previous studies did not control factors that could affect molecular adaptations induced by ESs. Among the others, hormones have been found to be the most important biomolecules affecting exercise adaptation and performance. New evidence suggests that adjusting PA with the body's natural hormonal biorhythms could maximize its benefits (Augsburger et al., 2025[2]). Testosterone and cortisol exhibit similar circadian patterns, with testosterone typically peaking between 8 and 9 a.m. and cortisol peaking between 7 and 8 a.m., which together optimize muscle-building and mobilization of energy during morning exercise sessions (Zar et al., 2021[28]). Human growth hormone (HGH) is released in bursts, with a particularly high release during deep sleep and in response to high-intensity exercise. During evening exercise, between 5 and 7 p.m., HGH release may be increased after exercise and coincide with natural performance peaks (Ritsche et al., 2014[17]). Catecholamines have higher levels in the late afternoon and initiate cardiovascular and metabolic responses to moderate-intensity exercise around 1 p.m. (Scheer et al., 2010[18]). In premenopausal women, estrogen (particularly estradiol) exhibits a slight circadian rhythm, with levels tending to be slightly higher from late morning to early noon, which can improve neuromuscular coordination, mood, and exercise tolerance during this period (Shao et al., 2021[20]). In addition, sex-specific variations in diurnal neuromuscular variability have been noted, with males exhibiting greater time-of-day variations in the development of force compared with females (Augsburger et al., 2025[2]). Based on these hormonal biorhythms, the best time to take ESs appears to be at 8 a.m., 1 p.m., and 6 p.m.

To highlight the effectiveness of the ESs program, it should be compared with previously approved traditional exercise programs. However, previous studies did not compare ESs with traditional exercise training or did not match the training load between ESs and standard exercise protocols. For example, Oliver J. Perkin (Perkin et al., 2019[14]), studied the effect of supervised resistance training (RT) compared to light home-based ESs. The RT group performed leg press, knee extensions, and curls three times weekly for four weeks at 60-75 % 1 repetition maximum (1RM). The ESs group performed brief bodyweight exercises such as squats, lunges, and stair-climbing three times daily during 14 days of low activity (< 1500 steps/day), with no monitoring of load or intensity.

Finally, we found that fewer than 6 % of publications followed up with the participants and examined the durability of adaptations. Exercise-induced molecular adaptations rapidly decline upon detraining, often within days to a week depending on the specific molecular factor, tissue, and training type studied (Lee et al., 2017[12]). This detraining effect manifests as reversed gene expression changes (e.g., mitochondrial biogenesis markers like PGC-1α return to baseline), reduced enzyme activities, and diminished physiological outcomes such as VO_2max_ or muscle strength (Lee et al., 2017[12]). However, these effects could be different between ESs and traditional exercise with snacks potentially offering more sustained signaling due to repeated daily stimuli (Islam et al., 2022[8]). While traditional training shows rapid molecular reversal, ESs could mitigate this via frequent activation of pathways like muscle protein synthesis, which persists longer with daily interruptions (Islam et al., 2022[8]). To address this research gap, future studies should include follow-up measurements of up to six months.

## Declaration

### Conflict of interest

The authors declare no competing interests.

### Artificial Intelligence (AI) - assisted technology

We did not use any AI during preparing this paper.

## Supplementary Material

Supplementary information
