# Supplementary material for: Current limitations of exercise snacks studies: A road map for the future
Source: EXCLI J. 2026 Jun 19;25:851–4. doi: 10.17179/excli2026-9491 (PMC13369880; doi:10.17179/excli2026-9491)
Supplement: Supplementary information [file EXCLI-25-851-s-001.pdf]

EXCLI Journal 2026;25:851-854 – ISSN 1611-2156
Received: April 15, 2026, accepted: May 22, 2026, published: June 19, 2026

Supplementary information to:

Letter to the editor:

CURRENT LIMITATIONS OF EXERCISE SNACKS STUDIES: A ROAD MAP FOR THE FUTURE

Fatemeh Sadat Masoudi[1], Farhad Daryanoosh[1], Kayvan Khoramipour[2*]

[1] Department of Sport Science, Faculty of Education and Psychology, Shiraz University, Shiraz 71946-84759, Iran
[2] i+HeALTH Strategic Research Group, Department of Health Sciences, Miguel de Cervantes European University (UEMC), 47012, Valladolid, Spain

* **Corresponding author:** Kayvan Khoramipour, i+HeALTH Strategic Research Group, Department of Health Sciences, Miguel de Cervantes European University (UEMC), 47012, Valladolid, Spain; E-mail: kkhoramipour@uemc.es

https://dx.doi.org/10.17179/excli2026-9491

This is an Open Access article distributed under the terms of the Creative Commons Attribution License (https://creativecommons.org/licenses/by/4.0/).

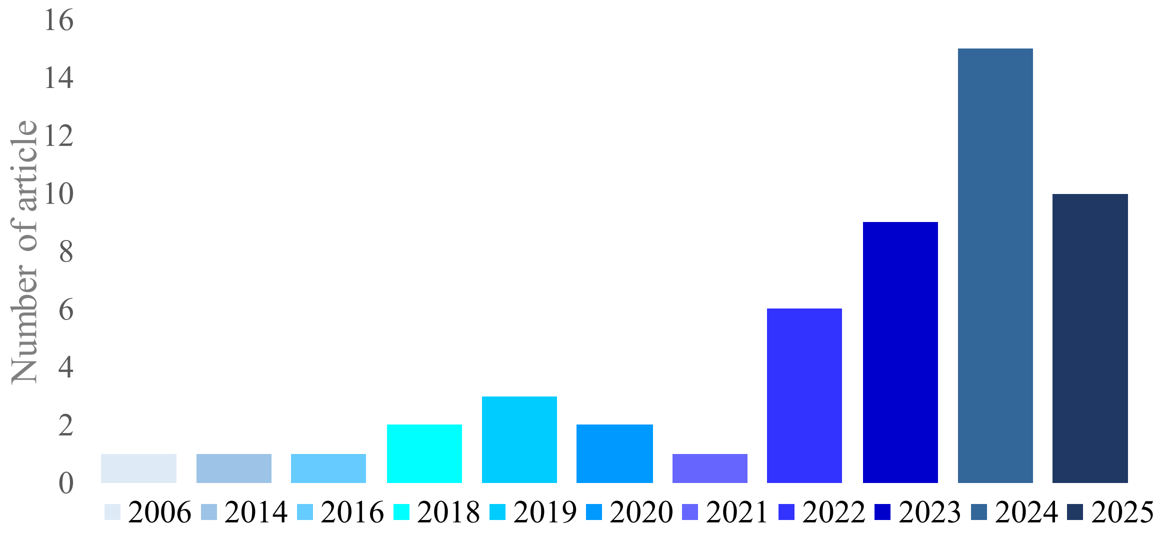

Supplementary Figure 1: Number of exercise snacks studies published each year; The number of published papers in 2025 is up to September 13th.
